# Transcranial electrical stimulation motor-evoked potentials in a spinal cord ischaemia rabbit model

**DOI:** 10.1186/s41016-019-0174-7

**Published:** 2019-12-05

**Authors:** Yucheng Lu, Baotao Lv, Qimin Song

**Affiliations:** 1 Central Laboratory, Linyi People’s Hospital, Shandong Province, Linyi, 276000 China; 2 Department of Radiology, Linyi People’s Hospital, Shandong Province, Linyi, 276000 China; 3 Department of Neurosurgery, Linyi People’s Hospital, Shandong Province, Linyi, 276000 China

**Keywords:** Spinal cord ischaemic injury, Motor-evoked potential, Amplitude, Rabbits

## Abstract

**Background:**

Spinal cord ischaemia animal models were established by selective ligation of the lumbar artery in a craniocaudal direction between the renal artery and the aortic bifurcation. Transcranial electrical stimulation motor-evoked potentials were measured to enable their use in future studies on spinal cord ischaemia protection.

**Methods:**

Thirty-three New Zealand rabbits were randomly divided into 6 groups. Transcranial electrical stimulation motor-evoked potentials were recorded before vascular ligation, 30 min after vascular ligation, and 2 days after vascular ligation. Motor functions were assessed after surgery and 2 days after vascular ligation. The specimens were taken 2 days after ligation for histopathologic observation.

**Results:**

With increased numbers of ligations, a transient extension of the latency became clear, but there were no significant differences in the statistical analysis. Analysis of variance after ligation at the same time in each group and *t* tests before and after ligation (*P* > 0.05) were not significant. One or 2 ligations did not cause spinal cord ischaemic damage. There were no significant differences before and after ligation for the amplitude (*P* > 0.05). With increased numbers of ligations, the amplitude before and after ligation was gradually reduced in the 3–5 ligation groups (*P* < 0.05).

**Conclusions:**

Ligation of segmental spinal cord vessels on 1 or 2 levels did not cause ischaemic damage. Spinal cord ischaemia was observed after 3, 4, or 5 ligations. The amplitude was more sensitive to spinal cord ischaemia than latency. Spinal cord function can be predicted by early changes in the amplitude.

## Background

Paraplegia and incontinence are serious complications of spine and spinal cord surgery caused by spinal cord injury [1]. Spinal cord ischaemic injury can be caused by intraoperative pulling or occlusion of blood vessels during spinal cord surgery. Understanding the characteristics and mechanisms of spinal cord ischaemia injuries can help us better protect spinal cord function and prevent spinal cord injury. Previous research has indicated that transcranial electrical stimulation motor-evoked potentials (Tes-MEPs) reflect the functional integrity of the motor pathway, especially of vulnerable motoneurons in the anterior horn of the spinal cord, and promptly respond to spinal cord ischaemic injury [2]. Motor-evoked potentials (MEPs) can reflect the function of the spinal cord in real time, as well as spinal cord blood supply. Intraoperative MEP monitoring has become an essential aspect of surgical technology. MEPs are sensitive to spinal cord ischaemia, specifically to motor function, and related to the degree of pathological damage to the spinal cord. Stable recordings document intact motor pathways and allow the surgeon to confidently proceed with the surgery. Recent research has focused mainly on obtaining a better understanding of the features of spinal cord blood supply in the hope of reducing complications [3]. Little is known about spinal cord functional changes after different levels of permanent spinal cord ischaemia and the compensatory mechanisms in response to sudden reductions in blood flow. To better understand the compensatory mechanisms of spinal cord ischaemia and the features of spinal cord ischaemic injury responsible for loss of spinal cord function, more research is needed to understand the anatomical features and dynamics of spinal cord blood supply and the reactions of the spinal cord to different levels of permanent spinal cord ischaemia.

In this experiment, we established different levels of permanent spinal cord ischaemia in rabbits by selective ligation of the lumbar artery in a craniocaudal direction between the renal artery and the aortic bifurcation. The functional integrity of the motor pathway and the adequacy of spinal cord blood supply were assessed using Tes-MEPs. The aim of this study was to study the change of Tes-MEPs after different levels of permanent spinal cord ischaemia.

## Methods

### Perioperative management

All experimental protocols of this study were approved by the Institutional Ethics Committee of Linyi People’s Hospital. All experimental animals were provided with adequate food and water and housed individually in metal cages at a temperature of 25–26 °C. The handling and use of laboratory animals conformed to the “Guidelines for animal experiments at Linyi People’s Hospital” and “Medical Laboratory Animal Management Regulations” and other relevant laws and regulations.

### Study design and management

Thirty-three New Zealand white rabbits, both male and female, weighing 3.0–3.5 kg were randomly selected for this experiment. The rabbits underwent different levels of serial lumbar artery ligation in a craniocaudal direction between the renal artery and the aortic bifurcation. The animals were divided into six experimental groups as follows: sham group, no ligation, *n* = 8; group 1, ligation of bilateral lumbar arteries at 1 level, *n* = 5; group 2, ligation of bilateral lumbar arteries at 2 level, *n* = 5; group 3, ligation of bilateral lumbar arteries at 3 level, *n* = 5; group 4, ligation of bilateral lumbar arteries at 4 level, *n* = 5; and group 5, ligation of bilateral lumbar arteries at 5 level, *n* = 5.

### Anaesthesia management

Intravenous access was established in the marginal ear vein. Anaesthesia (3% sodium pentobarbital) was infused through the marginal ear vein at 1 ml/kg and was maintained using 1/3 to 1/2 of the initial dose according to the response of animals during the experiment. Rabbits were intubated and connected to a respirator to control their breathing and provided with nitrous oxide and oxygen at a 2:1 ratio. Intravenous lactate ringer solution was infused according to the amount of bleeding. Body temperature was monitored continuously with a rectal thermometer during the experiment and was maintained between 38 °C and 39 °C with an electric blanket.

### Surgical technique

The experimental animals were put in the supine position. After sterile surgical preparation, additional local anaesthesia (0.5% lidocaine hydrochloride) was applied to the abdominal wall. A midline abdominal incision was made, and the bowels were taken out by turning them over to the left and covering them with wet, warm, and sterile gauze to reduce fluid and heat loss. The retroperitoneal cavity was opened and probed, the abdominal aorta between the renal artery and the aortic bifurcation was exposed, and the 5 lumbar arteries between the renal artery and the aortic bifurcation were exposed. The superior and inferior mesenteric arteries were untouched during the surgery.

### The experimental groups and the control group

In the experimental groups, a baseline Tes-MEP recording was recorded while anaesthesia conditions were stable. The lumbar arteries between the renal artery and the aortic bifurcation were ligated in a craniocaudal direction. Tes-MEPs were recorded 30 min and 2 days after ligation. The animals’ neurologic functions were assessed after awakening and 2 days after ligation. Spinal cords were quickly harvested 2 days after ligation for histopathologic observation. In the control group, stimulation with different intensities was used to induce Tes-MEPs to determine the most appropriate stimulation intensity. MEPs were recorded before and after the operation and every 30 min for 3 h.

### Monitoring technique for transcranial electrical stimulation motor-evoked potentials

The rabbits were placed in the prone position, and the skull was placed in a stereotaxic instrument. The scalp was infiltrated with 1% lidocaine. A 3-cm-long incision was cut in the scalp, exposing the skull. The sagittal and coronal sutures of the calvarium were exposed after the periosteum was removed. Stimulating electrodes were fixed on the skull, with the cathode placed in the C4 position and the anode in the C2 position [4]. The stimulating electrodes were connected to an EpochXP-2000 electrical stimulator (purchased from Axon system of America). Recording electrodes were made using silver acupuncture needles, placed in the subcutaneous area near the gastrocnemius muscle in the hind leg. Stimulation parameters included a stimulation train (three pulses, 120–130 V, 100 ms duration, and 2 ms interstimulus interval) that was used to elicit Tes-MEPs. Tes-MEPs were also recorded from the upper extremities to control for the procedure. Recording parameters were as follows: time base 100 ms, bandpass filter 30–3000 Hz, and amplified 5000 times. Tes-MEPs were recorded before the ligation, after 30 min, and 2 days after ligation. The baseline value was determined just prior to the start of lumbar artery ligation. The amplitude of Tes-MEPs was defined as the voltage from the most positive to the most negative component. After the baseline value of Tes-MEPs was recorded, prepared lumbar arteries were ligated in a craniocaudal direction at different levels.

### Evaluation of neurologic outcome

The motor function of the hind limbs of all rabbits was assessed after surgery and at 2 days after ligation. Tarlov’s score includes the following: 1, spastic paraplegia, cannot move; 2, paraparesis, slight movements; 3, paraparesis, powerful movements in the hind limbs but unable to stand; 4, able to stand but unable to walk; and 5, full recovery, normal walking function. Neurological examination was carried out at the same time by two investigators who were blinded to the groupings, who independently assessed the animals’ neurologic functions.

### Evaluation of pathologic outcome

All rabbits were killed with deep intravenously administered sodium pentobarbital anaesthesia (100 mg/kg) 2 days after surgery. The spinal cord between L2 and L4 was taken out and soaked in 10% paraformaldehyde/0.1 mol/L phosphate-buffered saline solution at 4 °C for 48 h. Sections were cut before being embedded in paraffin. The experimental slices were stained with haematoxylin-eosin and examined using light microscopy by neuropathologists blinded to the experimental groups. Destruction of spinal cord motor neurons in the anterior horn was quantified.

### Statistical analysis

Data are expressed as the means ± standard deviation. Multigroup variables were compared using one-way variance analysis. The paired *t* test was used for the analysis of Tes-MEP latency and amplitude before and after ligation in each experimental group. The paired *t* test was used for the analysis of motor function before and after ligation in each group. Analyses were implemented with SPSS 17.0 software. Difference with *P* values less than 0.05 was considered statistically significant.

## Results

### The comparisons of animal weight, heart rate, oesophageal temperatures, mean arterial pressure, and central venous preoperative pressure showed no differences between groups

**Fig. 1:**
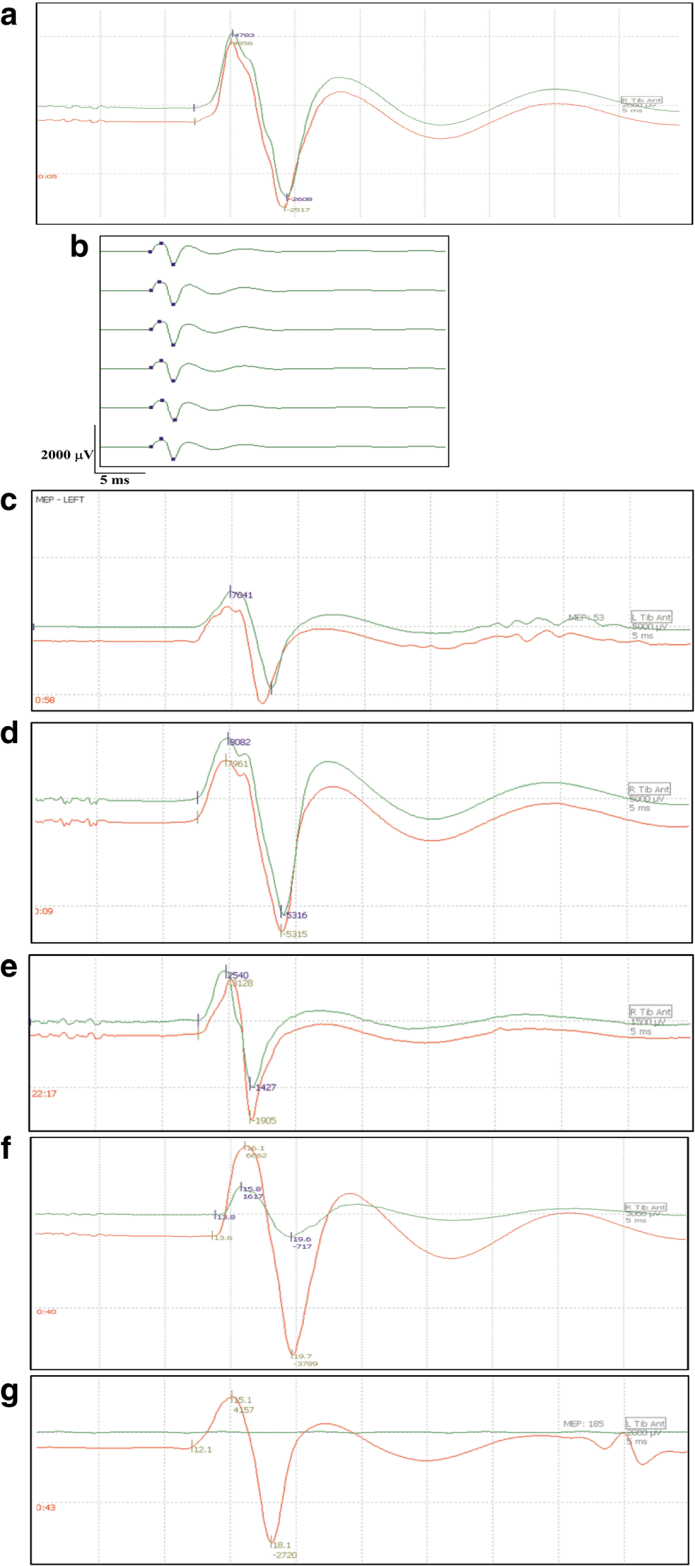
The amplitude and latency of Tes-MEPs were stable at different times after anaesthesia in the different groups. **a** Typical wave of transcranial electrical stimulation motor-evoked potentials. **b** Sham group. **c** Group 1. **d** Group 2. **e** Group 3. **f** Group 4. **g** Group 5. Tes-MEP latency is defined as the duration, in millisecond, from stimulation to the first progressive negative deflection. Tes-MEP amplitude is defined as peak-to-peak amplitude in microvolts (N1–P1). The red graphics indicate the positive and negative baseline waveform before ligation. The green graphics indicate the positive and negative baseline waveform after lumbar artery ligation

The 8 rabbits in the control group were used for exclusion of anaesthesia and surgery on evoked potentials and to determine the optimal stimulation intensity. Twenty-five rabbits were included in the spinal cord ischaemia groups. During the surgery, 3 rabbits were excluded for variation or intraoperative haemorrhage. After awakening, the rabbits were free to eat. Urine retention was not observed. The success rate of the model was 88%. Motor-evoked potentials recorded from the needle electrode in the gastrocnemius muscle in the hind leg were composed of a major initial negativity (upward deflection) followed by a positive deflection (downward deflection). Although the wave configuration and amplitude varied in different rabbits, the basic negative-positive waveform had good reproducibility and was high in amplitude. The typical MEP wave deflections were described as N1 and P1 (Fig. 1). Very few were described as N1, P1, and N2, or more. In all of the 33 animals, the latency was 12.27 ± 0.96 ms, and the amplitude was 6157.87 ± 2362.99 μV.

### The control groups

In the control groups, stimulation with different intensities was used to induce Tes-MEPs to determine the most appropriate stimulation intensity. With increasing stimulus intensity, the amplitude gradually increased and the latency gradually decreased. When the stimulation intensity was greater than 120 V, there was no change in the amplitude and latency. Thus, 130 V was used as the most appropriate stimulation intensity. There were no significant differences in Tes-MEP amplitude and latency at different time points after the rabbits were anaesthetised in the control groups (all, *P* > 0.05) (Table 1) (Fig. 1b). There were no significant differences in Tes-MEP amplitude and latency before and after the operation at different time points in the control groups (all, *P* > 0.05) (Table 2).

**Table 1:** The amplitude and latency of Tes-MEPs at different times after anaesthesia in the control group (mean ± standard deviation)

Time (min)	LAT (ms)^*^	AMP (μV)^*^
30	12.42 ± 1.26	5147.88 ± 2240.59
60	12.54 ± 1.24	5045.13 ± 2198.68
90	12.43 ± 1.20	5107.75 ± 2176.90
120	12.43 ± 1.20	5054.88 ± 2151.21
150	12.43 ± 1.15	5024.13 ± 2041.39
180	12.42 ± 1.16	5110.88 ± 2190.23

Analysis of variance

*P > 0.05

**Table 2:** The latency of Tes-MEPs at different times before and after surgery (mean ± standard deviation)

Group	Before ligation	30 min after ligation	2 days after ligation
Sham group (*n* = 8)	12.45 ± 1.17	12.45 ± 1.15	12.45 ± 1.17
Group 1 (*n* = 4)	11.20 ± 1.06	11.20 ± 1.06^★^	11.20 ± 1.06^★^
Group 2 (*n* = 5)	12.18 ± 0.52	12.16 ± 0.50^★^	12.18 ± 0.51^★^
Group 3 (*n* = 4)	12.92 ± 1.26	12.90 ± 1.27^★^	12.93 ± 1.26^★^
Group 4 (*n* = 4)	12.18 ± 0.61	13.28 ± 1.33^★^	12.30 ± 0.71^★^
Group 5 (*n* = 5)	12.48 ± 0.23	**–**	**–**

*t* test before ligation

^★^*P* > 0.05

### The experimental groups

#### Tes-MEP latency and amplitude

With increased numbers of ligations, we observed a transient extension of the latency, and there were no significant differences in the paired *t* test before and after ligation in each group (all, *P* > 0.05) (Table 2). The amplitude of the MEPs was expressed as a percentage of the values before ligation (baseline). The MEP amplitude was determined as the peak-to-peak amplitude of initial positive and negative waves.

There were no significant differences in the paired *t* test before and after ligation in 1- and 2-level ligation groups (*P* > 0.05). With an increase in the number of ligations, the ratio of the amplitude after and before ligation gradually decreased. Tes-MEPs were lost after 5 levels of ligation. All animals with Tes-MEP loss suffered postoperative paraplegia. There were significant differences in the paired *t* test before and after ligation in the 3–5-level ligation groups (Table 3) (Fig. 1c–g).

**Table 3:** The amplitude of Tes-MEPs at different times before and after surgery (mean ± standard deviation)

Time	Before ligation	30 min after ligation	2 days after ligation
Sham group (*n* = 8)	5267.50 ± 2264.04	4673.87 ± 1954.02	4926.38 ± 1964.48
Group 1 (*n* = 4)	100.00 ± 0.00	92.82 ± 5.60^★^	97.61 ± 2.07^★^
Group 2 (*n* = 5)	100.00 ± 0.00	92.82 ± 4.10^★^	98.09 ± 5.57^★^
Group 3 (*n* = 4)	100.00 ± 0.00	61.39 ± 2.12^△^	97.95 ± 1.65^★^
Group 4 (*n* = 4)	100.00 ± 0.00	21.63 ± 2.40^△^	40.38 ± 6.90^△^
Group 5 (*n* = 5)	100.00 ± 0.00	1.09 ± 2.44^△^	2.15 ± 4.82^△^

*t* test before ligation

^★^*P* > 0.05

^△^*P* < 0.01

#### Functional impairments

There were no significant differences in motor function before and after surgery in the 1 and 2 ligation groups (*P* > 0.05). There were significant differences in motor function before and after surgery in the 4 and 5 ligation groups (*P* < 0.05). In the 3 ligation group, there were significant differences after anaesthesia awake (*P* < 0.05) but no significant differences 2 days after ligation (*P* > 0.05) (Table 4).

**Table 4:** Functional evaluations before and after surgery (mean ± standard deviation)

Time	Before ligation	Anaesthesia awake	2 days after ligation
Sham group (*n* = 8)	5.00 ± 0.00	5.00 ± 0.00^★^	5.00 ± 0.00^★^
Group 1 (*n* = 4)	5.00 ± 0.00	5.00 ± 0.00^★^	5.00 ± 0.00^★^
Group 2 (*n* = 5)	5.00 ± 0.00	5.00 ± 0.00^★^	5.00 ± 0.00^★^
Group 3 (*n* = 4)	5.00 ± 0.00	3.75 ± 0.50^△^	5.00 ± 0.00^★^
Group 4 (*n* = 4)	5.00 ± 0.00	1.75 ± 0.96^△^	2.50 ± 0.58^△^
Group 5 (*n* = 5)	5.00 ± 0.00	0.00 ± 0.00^△^	0.20 ± 0.45^△^

*t* test before ligation

^★^*P* > 0.05

^△^*P* < 0.05

#### Histologic assessment

**Fig. 2:**
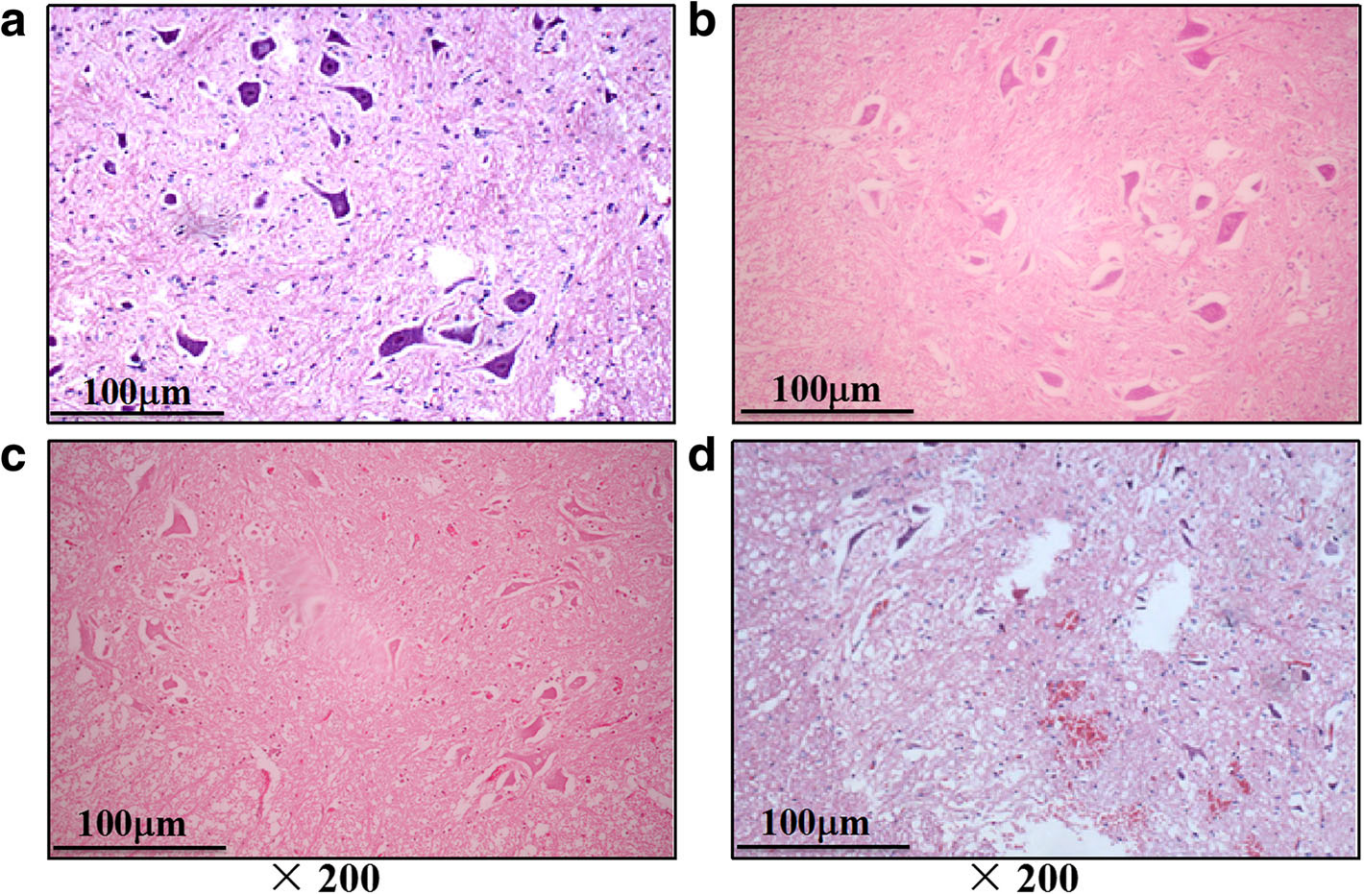
HE (haematoxylin-eosin) staining of spinal cord sections at the 2 days after operation. **a** No sign of neurnal damage was observed in the sham group. **b** Decrease of the cell volume of neuron and karyopyknosis were observed in group 3. **c** Reduction of the number of neuron and vacuolation of grey matter were observed in Group 4. **d** Apoptotic and necrotic motoneurons in the ventral horn were observed in group 5

In the sham group, group 1, and group 2, the spinal cord was normal, with many intact motoneurons in the anterior spinal horn (Fig. 2a). Spinal cord ischaemic injury was observed in the motoneurons of the anterior spinal horn and grey matter in the 3-, 4-, and 5-level ligation groups. We observed neuronal necrosis, with typical loss of cytoplasmic structures and eosinophilic cytoplasm, as well as indicators of neuronal apoptosis, such as apoptotic bodies, chromatin condensation, shrinkage, and nuclear fragmentation. The extent of spinal cord ischaemic injury was proportional to the number of ligations in group 3, group 4, and group 5. The spinal cord suffered mild to moderate injury in group 3, group 4, and group 5 (Fig. 2b–d).

We found a significant relationship between amplitude changes 30 min after ligation in the 3, 4, and 5 ligation groups and postsurgery motor function. The correlation was significant using a 2-tailed test; the *P* < 0.001 and the Pearson’s correlation coefficient was 0.977.

## Discussion

During thoracic and thoracoabdominal aortic aneurysm repair surgery involving serial intercostal and lumbar artery ligation, neurophysiologic monitoring is commonly used to guide the operative strategy and prevent of paraplegia. During the resection of intramedullary spinal cord tumours, intraoperative neurophysiological monitoring is also used to assess the status of the spinal cord. Interpretation and recording of MEPs is fast. Postoperative clinical motor functions correlate with intraoperative MEPs results. Intraoperative neurophysiological monitoring has become an integral part of spine surgery because it can assess spinal cord function and help predict the prognosis [5]. Rabbit models are essential to design safety and protective measures for clinical applications for preventing postoperative paraplegia and incontinence [6]. The earliest spinal cord ischaemia injury animal models were established using abdominal aortic ligation below the renal artery. Many problems occur in this animal model, such as intestinal rupture or coagulopathy [7]. Lips et al. clamped the lumbar arteries in a caudal-to-cranial direction to cause spinal cord ischaemia in pigs [8]. Wadouh et al. occluded all of the lumbar arteries from L1 to S1 in pigs [9]. In our study, we find that the left and right lumbar arteries in rabbits originate from the aorta as a common trunk. This structure is particularly suitable for an animal model of spinal cord ischaemia using ligation.

The spinal cord depends upon an extensive and redundant blood supply. Nambu et al. reported that blood flow to the spinal cord remained three quarters of control values in dogs after ligation of the lumbar arteries at 3 consecutive levels [10]. Ueda et al. showed that spinal cord function was normal and blood flow to the spinal cord was approximately 85% of the control value after ligation of the segmental arteries at 3 consecutive levels [11]. Schurink et al. reported that no signs of spinal cord motor dysfunction were found in 6 patients who had ligation of the segmental arteries because the surgery requires 9.8 ± 3.2 segmental arteries [12]. In our study, we found that 1–2 levels of lumbar artery ligation did not cause ischaemic spinal cord damage. With increasing numbers of ligations, the Tes-MEP amplitude gradually reduced in the 3–5 ligation groups (*P* < 0.05). Spinal cord ischaemic injury was observed in the motoneurons of the anterior spinal horn and grey matter in the 3-, 4-, and 5-level ligation groups, which included neuronal necrosis and neuronal apoptosis.

The electrophysiological monitoring techniques used in spinal surgery rely on somatosensory-evoked potentials (SEPs) or MEPs or electromyography [13]. SEPs have been used to monitor spinal cord function during spinal surgery for a long time [14]. SEP monitoring is non-invasive and useful for detecting spinal cord structural injury or spinal cord ischaemic injury [15]. However, SEP monitoring has some disadvantages. SEP waveform cannot be recorded instantly in real time because every waveform signal is so small that the monitoring signals must be averaged to create an averaged SEP wave. The wave indicates only the functional integrity of the sensory conduction pathway, which is located in the dorsal portion of the spinal cord and is supplied by the posterior spinal artery. Thus, the SEP wave directly reflects the functional integrity of sensory pathway of spinal dorsal horn. False-negative results of spinal cord motor dysfunction have been reported. A more reliable and sensitive method of monitoring intraoperative motor function must be used to detect intraoperative spinal cord injury to prevent paralysis and incontinence.

Tes-MEPs reflect the functional integrity of motor pathways [16, 17], especially those sensitive to ischaemic injury, like the motor neurons in the anterior spinal horn. Tes-MEPs immediately react to spinal cord injury. Tes-MEP monitoring is a physiologically and anatomically sound means of detecting functional motor injury of the spinal cord. Detection of transient and acute spinal cord ischaemic injury with MEPs occurs quite quickly. Intraoperative monitoring of MEP has proven real-time feedback, clinical correlation, and reversibility, allowing for protective measures to be taken before a spinal cord injury becomes irreversible. Recently, Tes-MEPs were used for monitoring of spinal surgery because its amplitude is high enough and it is non-invasive and sensitive to spinal cord injury [18]. The advantages of MEPs have been confirmed in clinical surgery and animal experiments. Real-time information about regional spinal cord ischaemia can guide intraoperative management and reduce the risk of paraplegia [19]. Monitoring of MEPs is now frequently used during spinal cord surgery [20]. Patients with intact MEPs usually awaken without motor dysfunction [21]. An amplitude decreased to 50% of the baseline in surgery has been used as a warning standard. In our study, reproducible Tes-MEPs were recorded in all experimental animals. There was no spinal cord ischaemic injury in the control group and 1- or 2-level ligation groups. With an increased number of lumbar arteries ligated, the degree of spinal cord ischaemia gradually increased and the amplitude lowered quite soon after lumbar artery ligation. In the 3-level ligation group, the wave amplitude of the Tes-MEPs decreased to 61.39% ± 2.12% 30 min after ligation. Functional evaluation was normal 2 days after ligation. In the 4-level ligation group, the wave amplitude of the Tes-MEPs decreased to 21.63% ± 2.40% 30 min after ligation. Functional evaluation was abnormal 2 days after ligation. In the 5-level ligation group, the wave amplitude of the Tes-MEPs completely disappeared and the functional evaluation showed the animals to be completely paralysed. Therefore, the 50% of the baseline used as the warning standard was supported in our study.

Although MEP intraoperative monitoring has been successfully used to guide surgery, prevent postoperative neurologic deficits, and detect spinal cord ischaemia [22], it has limitations that have prevented it from being widely used [23]. For example, the muscle relaxants that are part of the usual surgical drugs interfere with MEP intraoperative monitoring. Thus, the technique is not widely used [24]. More animal experiments are needed to make intraoperative monitoring widely used in clinical surgery.

## Conclusions

In our experiment, different levels of permanent spinal cord ischaemia were induced using selective ligation of the lumbar artery in a craniocaudal direction between the renal artery and the aortic bifurcation. In our study, we showed that Tes-MEPs reflect transmission in the spinal motor pathway and quickly detect spinal cord ischaemic injury. Spinal cord motor function can be assessed by early changes to the MEP amplitude.

## Data Availability

The datasets used and analysed during the current study are available from the corresponding author on reasonable request.

## Abbreviations

MEPs: Motor-evoked potentials
SEPs: Somatosensory-evoked potentials
Tes-MEPs: Transcranial electrical stimulation motor-evoked potentials
